# A pedicled anterolateral thigh flap for reconstruction of abdominal wall soft tissue defect in the absence of microsurgical services- case report

**DOI:** 10.1016/j.ijscr.2025.111266

**Published:** 2025-04-07

**Authors:** Metasebia W. Abebe, Getachew T. Abate

**Affiliations:** Metasebia W. Abebe, Plastic and reconstructive Surgeon, Department of Plastic and Reconstructive Surgery, St. Paul's Hospital Millennium Medical College, Addis Ababa, Ethiopia; Getachew T. Abate, Plastic and Reconstructive Surgeon, St. Paul's Hospital Millennium Medical College, Addis Ababa, Ethiopia

**Keywords:** ALT flap, Pedicled flap, Soft tissue defects, Abdominal wall, Case report

## Abstract

**Introduction:**

The anterolateral thigh (ALT) flap is a known work horse flap for coverage of range of soft tissue defects. It is utilized both as pedicled and free flap. It is a versatile flap that covers large area of cutaneous territory and which can be augmented with the vastus lateralis muscle for bulk or with the tensor fascia for fascia reconstruction. It has a long, wide caliber pedicle which gives it the freedom to be mobilized proximally to the level of the xiphoid process of the sternum and distally to the level of the knee.

**Case presentation:**

We have presented here a case of a 19-year old man with blast injury to the abdomen where we have used pedicled antero-lateral thigh flap to cover a large abdominal wall defect.

**Discussion:**

The anterolateral thigh flap is a workhorse flap in reconstructive surgery that can be used as a durable coverage for abdominal wall defects -to protect the abdominal viscera and avoid loss of domain and other disruptions of normal physiology.

**Conclusion:**

The pedicled anterolateral thigh (ALT) flap was performed under 3.5× loupe magnification, demonstrating the feasibility of using this technique for abdominal wall defects in settings without full microsurgical services. While the procedure can be performed without an operating microscope, it still requires basic microsurgical equipment—such as loupes—and fundamental microsurgical skills, underscoring the importance of proficiency in these essential techniques.

## Highlights

• Abdominal wall defects can be full thickness defects resulting in loss of domain.
• Abdominal wall defects result from infectious processes, trauma or tumor resections.
• Reconstruction restores domain, contributes to posture and physiologic functions of the body.
• Pedicled anterolateral thigh flap can be used for abdominal wall reconstruction.
• Pedicled ALT flap is an excellent alternative in the absence of microsurgical services.

## 1 Introduction

The anterolateral thigh (ALT) flap was first described by Song et al. [1] in 1984 as a septocutaneous perforator flap supplied by the descending branch of the lateral circumflex femoral artery (LCFA). It is widely regarded as a “workhorse flap” in reconstructive surgery due to its numerous advantages. These include the ample length and suitable diameter of its vascular pedicle, which facilitates microvascular anastomosis. The flap's versatility stems from its large cutaneous territory with high-quality skin, its shape adjustability, and the ability to achieve aesthetically hidden donor site scars. Depending on the specific defect requirements, the ALT flap can be raised as a fasciocutaneous, adipofascial, or myocutaneous flap [[1], [2], [3], [4], [5], [6]]. The flap's pedicle can be derived from either septocutaneous or musculocutaneous perforators originating from the descending branch of the LCFA. To optimize outcomes, preservation of the vena comitantes, lateral femoral vein, and the anterior or lateral femoral cutaneous nerves is recommended to ensure proper venous drainage and maintain sensation [[1], [2], [3],^5^].

The length of the robust vascular pedicle of descending branch of lateral circumflex femoral artery can be increased to 15 cm (it ranges from 8.5 cm to 14 cm) by dissecting up to its origin from profunda femoris [1,2,7,8]. Majority of the perforators are located in the middle one-third of the line running from the anterosuperior iliac spine to the superolateral border of the patella [9].

The use of free ALT flap in various sites as scalp, neck, face/oral cavity, pharyngoesophageal, breast, chest and abdominal wall as well as extremity reconstruction has been well described [2]. Its versatility in pedicled form is reflected in its use in coverage of abdominal wall defects up to 8 cm above the umbilicus reaching the xiphoid [3,6,10,11] groin, perineum, contralateral lower abdomen and lower back, inguinal area, trochanteric and hip defects and even defects of the knee based on distal perforators that anastomose to lateral superior genicular artery or the profunda femoris artery perforators [2,5,7,8,10].

Though the use of the anterolateral thigh (ALT) free flap is well described, reports on the pedicled ALT flap are relatively scarce in the literature [12]**.** We have demonstrated its crucial role in covering large abdominal wall defects. This flap can be reliably raised using 3.5× loupe magnification, making it a valuable option in resource-limited settings without access to full microsurgical services. Importantly, the successful execution of this complex procedure depends not only on the technique but also on the surgeon's expertise and experience. Here, we discuss a case where a pedicled ALT flap was used to reconstruct an abdominal wall defect resulting from a blast injury in a young soldier in a referral teaching hospital where there is no operating microscope, highlighting how experienced surgeons can perform intricate surgeries even in resource-constrained environments.

This work has been reported in line with the SCARE criteria [13].

## 2 Case

A 19 – year - old male patient who sustained blast injury to his abdomen. He was treated at a nearby hospital where he underwent exploratory laparotomy. Their intraoperative findings were transverse colon perforation with ruptured caecum that was managed with a right hemi colectomy with end ileostomy. He was referred to our hospital on his second post-operative date for subsequent management.

**Fig. 1:**
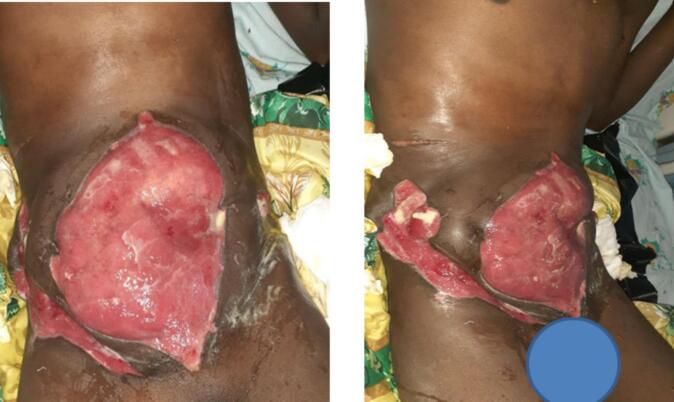
The abdominal wall defect extending form the supra-umbilical area to the right subcostal region with exposed bowel loops and thin layer of granulation tissue and stoma on the left lower quadrant.

At presentation, he was diagnosed with necrotizing soft tissue infection of the abdominal wall with malodorous purulent exudate. On abdominal examination, he had huge abdominal wall full thickness defect measuring 18 cm × 14 cm with a thin bridge of skin in between. The defect extended from supra pubic area to the right subcostal region. His bowels were eviscerated and he had stoma on left lower quadrant of the abdomen (Fig. 1). Laboratory examinations showed normal hemoglobin level of 10 g/dl, leukocytosis with white blood cell counts 18,000/μL with neutrophil of 82 %, hypokalemia with potassium level of 2.07 mmol/l and hypoalbuminemia with albumin level of 1.8 g/dl but other parameters of renal and liver function panels were normal.

**Fig. 2:**
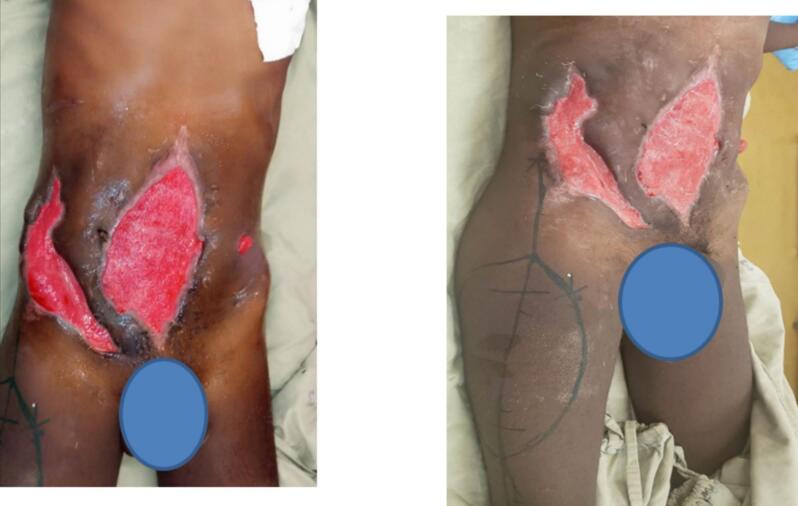
Well granulated 16 cm × 12 cm anterior abdominal wall wound, left side stoma and markings of the planned pedicled ALT flap on the right thigh.

The Infection was treated with multiple debridement and washout, antibiotic coverage with Ceftazidime and Vancomycin for broad spectrum coverage and daily wound care with vaseline gauze prepared at the hospital. Hemoglobin and potassium was corrected over his stay in the hospital. He continued on daily wound care and high calorie, high protein diet for a total of four weeks (Fig. 2).

The abdominal wall defect was covered with granulation tissue and the wound had contracted to 16 cm × 12 cm defect, but a stable soft tissue cover was needed since there was a plan of reversing the ileostomy. Skin graft as an option of soft tissue cover was not considered since it would make the surgery for ileostomy reversal very difficult. After careful consideration and discussion with the colorectal surgery team and the patient, it was decided to do a pedicled anterolateral thigh flap for a durable coverage of the defect and ease of stoma reversal.

**Fig. 3:**
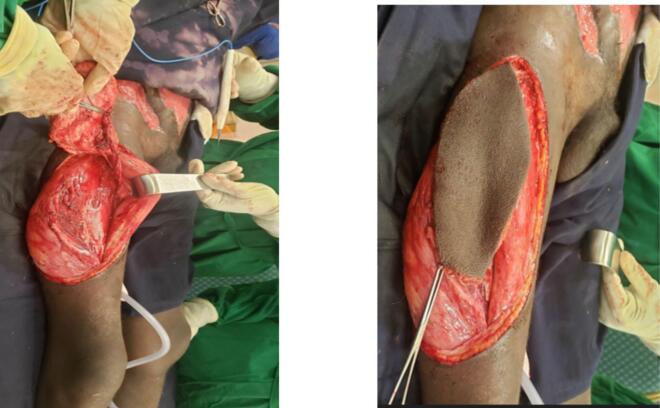
Intraoperative pictures of the pedicled ALT flap.

Under general anesthesia and supine positioning, an ALT flap was designed over the right thigh using a template from the defect and centering it on the middle third of the line drawn from the anterior superior iliac spine to the superolateral corner of the patella. The flap was raised as a fasciocutaneous flap from medial to lateral identifying the pedicle within the intermuscluar septum between the rectus femoris and vastus lateralis. The LCFA was dissected up to its origin at the profunda femoris artery. All fascia attachments released and flap fully mobilized to allow it to reach to the proximal end of the abdominal defect (Fig. 3).

**Fig. 4:**
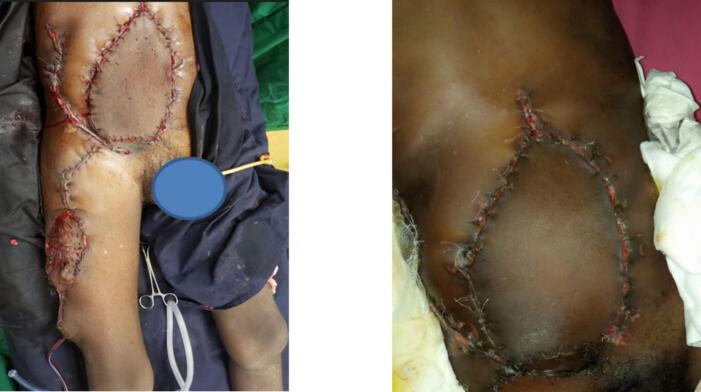
Immediate and 48-h post-operative picture of the flap inset over the abdominal defect and skin graft used to cover the donor site.

The islanded skin flap was undermined and mobilized laterally and sutured to the remaining abdominal wall tissue on the right side while the flap was inset to the central abdominal wall defect over a drain. The donor site was partially closed but a split thickness skin graft was used in an area where primary closure was not possible (Fig. 4).

**Fig. 5:**
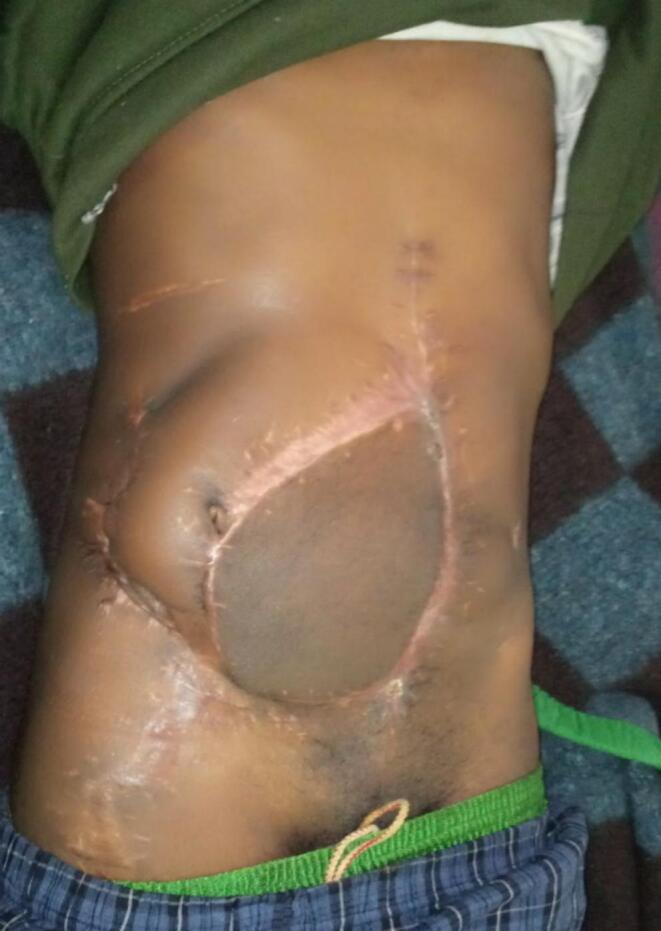
Post-operative month-6 with well healed flap and reversed ileostomy.

The patient was then discharged with stoma for further nutritional rehabilitation after which he was taken back for surgery by the colorectal team two months after the abdominal wall reconstruction for ileostomy reversal. Both surgeries were successful but the umbilicus was displaced laterally to the right side but the patient deferred any further surgery to create neoumbilicus for better appearance at his 6-month follow up visit (Fig. 5).

## 3 Discussion

The abdominal wall soft tissue defects range from full thickness skin defects to defects involving the abdominal muscle and fascia. Most soft tissue defects are commonly caused by peritonitis, laparotomies, trauma or en bloc resection of tumors [14,15]. Treatment options for complex abdominal wall defects include component separation, partition technique, flap coverage, and more recently acellular dermal matrix.

When it comes to flap coverage of a complex abdominal wall defect, the ALT flap which was initially described by the Song et al. [1] in 1984 as a septo cutaneous perforator flap, is a versatile flap supplied mostly by musculo- cutaneous perforators (80–87 %) and septo-cutaneous perforators (13–20 %) is widely used as a free flap for head and neck reconstruction, trunk and extremity defects [2,6,16].

As a pedicled flap, ALT can be used to cover ipsilateral [4,8] and contralateral [7] groin defects, abdominal wall [5,8,11,14]. The pedicled ALT flap has less cumbersome dissection of pedicle and has shorter duration of surgery compared to free ALT. Pedicle dissection can be done with magnifying surgical loupes.

We present a case of a complex abdominal wall defect reconstructed using an ipsilateral pedicled anterolateral thigh (ALT) flap. The reconstruction posed significant challenges due to the extensive open abdominal wound, which required prolonged debridement and cleaning, placing the patient in an extended catabolic state. In resource-limited settings where operating microscopes are unavailable, the pedicled ALT flap offers an excellent alternative for reconstructing complex abdominal wall defects and restoring domain—provided there is adequate surgical expertise, effective infection control, and thorough patient optimization.

## CRediT authorship contribution statement

Both Metasebia W Abebe and Getachew T Abate were involved in the full course of management of the patient and equally contributed in the preparation of the manuscript.

## Consent

Written informed consent was obtained from the patient for publication of this case report and accompanying images. A copy of the written consent is available for review by the Editor-in-Chief of this journal on request.

## Ethical approval

IRB approval is not needed for case reports in Saint Paul Hospital Millennium Medical College.

## Guarantor

Metasebia W. Abebe, Getachew T. Abate

## Funding

There was no funding related to this manuscript.

## Declaration of competing interest

Both authors declare that they have no conflict of interest to disclose with regards to this manuscript.
